# Polypropylene/Polyvinyl Alcohol/Metal-Organic Framework-Based Melt-Blown Electrospun Composite Membranes for Highly Efficient Filtration of PM_2.5_

**DOI:** 10.3390/nano10102025

**Published:** 2020-10-14

**Authors:** Ting-Ting Li, Yujia Fan, Xixi Cen, Yi Wang, Bing-Chiuan Shiu, Hai-Tao Ren, Hao-Kai Peng, Qian Jiang, Ching-Wen Lou, Jia-Horng Lin

**Affiliations:** 1 Innovation Platform of Intelligent and Energy-Saving Textiles, School of Textiles Science and Engineering, Tiangong University, Tianjin 300387, China; tingtingli@tiangong.edu.cn (T.-T.L.); 18332171860@163.com (Y.F.); 15858136960@163.com (X.C.); w17849235881@163.com (Y.W.); renhaitaomail@163.com (H.-T.R.); skyphk@163.com (H.-K.P.); gigglejq@hotmail.com (Q.J.); 2 State Key Laboratory of Separation Membranes and Membrane Processes, Tiangong University, Tianjin 300387, China; 3 Ocean College, Minjiang University, Fuzhou 350108, China; 4 Department of Bioinformatics and Medical Engineering, Asia University, Taichung 41354, Taiwan; 5 Department of Medical Research, China Medical University Hospital, China Medical University, Taichung 40402, Taiwan; 6 Fujian Key Laboratory of Novel Functional Textile Fibers and Materials, Minjiang University, Fuzhou 350108, China; 7 Laboratory of Fiber Application and Manufacturing, Department of Fiber and Composite Materials, Feng Chia University, Taichung 40724, Taiwan; 8 School of Chinese Medicine, China Medical University, Taichung 40402, Taiwan

**Keywords:** PM_2.5_, zeolite imidazole framework-8 (ZIF-8), melt-blown, electrospinning

## Abstract

Particulate matter 2.5 (PM_2.5_) has become a public hazard to people’s lives and health. Traditional melt-blown membranes cannot filter dangerous particles due to their limited diameter, and ultra-fine electrospinning fibers are vulnerable to external forces. Therefore, creating highly efficient air filters by using an innovative technique and structure has become necessary. In this study, a combination of polypropylene (PP) melt-blown and polyvinyl alcohol (PVA)/zeolite imidazole frameworks-8 (ZIF-8) electrospinning technique is employed to construct a PP/PVA/ZIF-8 membrane with a hierarchical fibrous structure. The synergistic effect of hierarchical fibrous structure and ZIF-8 effectively captures PM_2.5_. The PP/PVA composite membrane loaded with 2.5% loading ZIF-8 has an average filtration efficacy reaching as high as 96.5% for PM_2.5_ and quality factor (Q*_f_*) of 0.099 Pa^−1^. The resultant membrane resists 33.34 N tensile strength and has a low pressure drop, excellent filtration efficiency, and mechanical strength. This work presents a facile preparation method that is suitable for mass production and the application of membranes to be used as air filters for highly efficient filtration of PM_2.5_.

## 1. Introduction 

In recent years, air pollution has exacerbated particularly due to the presence of suspended minor particles that have aerodynamic diameter smaller or equal to 25 µm (i.e., particulate matter, PM_2.5_) [1,2,3] and are prone to carry hazardous substances, such as heavy metals and microorganisms. These substances can trigger diverse diseases and threaten human health [4,5,6,7,8]. In this regard, highly efficient air filters for PM_2.5_ filtration should be developed to address this imperative [9]. At present, traditional fiber filtration is commonly used in cleansing air or in our daily lives. Filtration is an effective approach used to remedy on-site PM pollution at the source [10]. Fibrous filters can be classified into spun-bonded filters, needle-punched filters, melt blown filters, and electrospun filters [11]. Filters capture particles by diffusion, interception, impaction, and gravitational settling [12]. Spun-bonded and needle-punched filters are excluded from the use of filters because they have a large diameter. 

Melt-blown membranes are the most commonly used in advanced air filters because of its high yield, high strength, and narrow pore size distribution [13]. Melt-blown fibers with a small diameter are crossed and distributed evenly to obtain a large specific surface area. The high filter efficacy and low pressure drop resistance of melt-blown membranes render them a priority in filter material selection. Fiber fineness should be reduced to enhance the filtration efficacy for ultra-small particles. However, performing the fiber formation technique of thinning fibers to the greatest extent is challenging, so electrospinning technology is incorporated in production. This technology is conducted in a high-voltage electric field to provide the polymer solution or melt with electricity. When the electric field is sufficient to overcome the surface tension of electrospinning solution, polymers can be induced, ejected, and expanded to form nanofibers [14]. Overall, electrospinning technology is a simple approach used to produce continuous non-woven nanofiber membranes with a large specific area, random aliment, high air permeability, and good perviousness [15,16,17]. Composites membranes are defined as the merging of inorganic and organic materials to obtain enhanced membranes by joining the strengths of materials [18]. This method improves the reduction in the fineness of melt-blown fibers and realizes an even distribution of zeolite imidazole frameworks-8 (ZIF-8) in the polypropylene (PP) melt-blown membranes. The efficiency of filter matrices can be enhanced through the deposition of an electrospun fiber layer on melt-blown fibers [19]. For example, in 2017, Han Joo Kim et al. produced silver nanoparticle-incorporated bi-layered electrospun melt-blown micro/nanofibrous products with improved filtration efficiency [19]. 

Metal-organic frameworks (MOFs) are an emerging class of porous crystalline materials with high porosity [20] and large surface area [21]. MOFs have been increasingly used in gas storage and separation fields [22]. ZIF-8 plays an important role in MOFs because its porous structure, smaller tunnels, and surface area are better than those of zeolite. The tunnel structure of ZIF-8 can be adjusted according to practical uses. ZIF-8 has a cage opening diameter of 0.34 nm, a surface area of 1900 m^2^/g, and thermal stability at 450 °C, indicating its specific resilience, extremely high thermal stability, chemical stability, and high micro-pore porosity [23,24]. ZIF-8 is also a good adsorbent. ZIF-8 is a kind of metal-organic framework, which not only can capture PM_2.5_ efficiently but also possesses excellent chemical and thermal stability [25]. Evenly loaded ZIF-8 nanocrystals provide compatible pore diameter. Furthermore, the majority of functional groups over ZIF-8 possess acid/alkali properties, which can interact with other acid/alkali functional groups and enable the membrane for adsorption or separation in gas and catalyst fields. For example, in 2019, Ma et al. prepared Ag-MOFs@CNF@ZIF-8 biodegradable cellulose-based filters. The PM_2.5_ filtration efficiency of the composite filters of pure cellulose increased from 44% to 94.30%, and the pressure drop increased from 19 Pa to 158 Pa [26]. In 2018, Su et al. prepared multifunctional CFs@ZIF-8 filters that demonstrated considerably high filtration efficiency, but the pressure drop increased from 197.5 Pa to 680.5 Pa [27]. Meanwhile, the electrospun fiber grafted with a large specific surface area composite can be used in the absorption field [28,29]. The presence of ZIF-8 improves the filtration efficiency and thus resolves the pressure drop issue. As such, filters should be improved in terms of high filtration rate and low pressure drop. The micro-nano structure of membranes is also expected to be applied to microfiltration, ultrafiltration, nanofiltration aspects [30]. 

In this study ZIF-8, polyvinyl alcohol (PVA) membrane, and PP membrane are combined via electrospinning and melt-blown technology to improve mechanical properties and filtration performance. Combined with the advantages of melt-blown and electrospinning, the addition of ZIF-8 with excellent structure and large specific surface area can improve the adsorption efficiency of the membrane. This work evaluated the filtration efficacy and analyzed the influences of the content of ZIF-8 and determined the optimal melt-blown filter materials for PM_2.5_.

## 2. Experiment 

### 2.1. Materials 

The polypropylene (PP, HP563S, Daelim Corporation, Seoul, Korea) had a density of 0.9 g/cm^3^, a melt flow rate (MFR) of 35 g/10 min, a shrinkage of 1.3–1.7%, and a tensile yield strength of 29 MPa. Zinc nitrate Zn(NO_3_)_2_·6H_2_O, (10196-18-6) was purchased from Tianjin city Damao Chemical Reagent Factory, China. 2-Methylimidazole (693-98-1) was purchased from Aladdin Reagent Company, Shanghai, China. Methanol (CH_3_OH, Chemical Reagent Factory, Guangzhou, China) had an analytical reagent (AR) grade. Polyvinyl alcohol (9002-89-5) was purchased from Aladdin Reagent Company, Shanghai, China.

### 2.2. Preparation of Polypropylene (PP) Melt-Blown Membranes 

PP membranes were prepared by a single screw melt-blown apparatus (Shengruiyuan Machinery Technology, Tianjin, China). PP particles were melted and pushed forward through the pipeline, metering pump, die, and eventually the mold by the shearing force of the extruder. A hot wind tube blew and shaped PP into fibers, which were then collected over the collection mesh to form PP melt-blown membranes. The mold had a specification of 9 holes/cm and diameter of 0.5 mm. The temperatures and parameters of the melt blowing apparatus were shown in Table 1 and Table 2. 

### 2.3. Synthesis of Zeolite Imidazole Frameworks-8 (ZIF-8)

An electronic balance (FA2104, Deante Sensor Technology Co., Ltd., Tianjin, China) was used to prepare 3 mmol of Zn (NO_3_)_2_·6H_2_O, which was then dissolved in 1050 mmol absolute methanol. Next, 15 mmol 2-methylimidazole was dissolved in 33.6 mL of methyl alcohol and mixed at room temperature for 30 min. The 2-methylimidazole absolute methanol mixture was infused into the zinc nitrate hexahydrate/absolute methanol mixture for 3 h by using a magnetic stirrer (Wiggens, Berlin, Germany). The mixture was kept still for 12 h. The mixture was processed with centrifugation, rinsed three times by using absolute methanol, and dried at 70 °C in a vacuum drying oven (Shanghai Boxun Industry& Commerce Co., Ltd., Shanghai, China).

### 2.4. Preparation of PP/Polyvinyl Alcohol (PVA)/ZIF-8 Melt-Blown Electrospun Composite Membranes 

**Figure 1:**
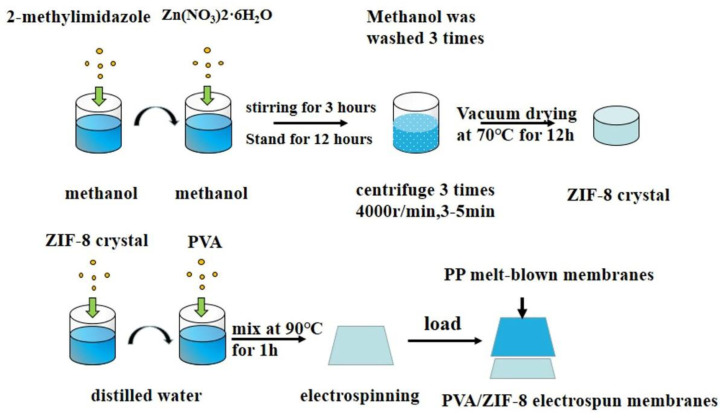
Flow chart of preparation of polypropylene/polyvinyl alcohol/zeolite imidazole frameworks-8 (PP/PVA/ZIF-8) melt-blown electrospun composite membranes.

Figure 1 shows the preparation of PP/PVA/ZIF-8 melt-blown electrospun composite membranes. First, 10% of PVA was dissolved in 65% of distilled water and stirred at 90 °C by using a magnetic mixer for 3 h. Distilled water (25%) was then added with 0%, 1.25%, 2.5%, 3.75%, and 5% ZIF-8 and processed at 50 °C with an ultrasonic instrument for 1 h. The ZIF-8 solution and PVA solution were mixed for 1 h and kept still for cooling to obtain PVA/ZIF-8 solvent. Afterwards, two medical syringes were used to draw 4 mL of the solvent for electrospinning. The solvent was loaded over PP melt-blown membranes. The denotations and specifications of different PP/PVA/ZIF-8 melt-blown electrospun composite membranes are presented in Table 3 and Table 4. 

### 2.5. Measurements

The filtration efficacy of PP/PVA/ZIF-8 melt-blown membranes was measured using an automated filter tester (TOPAS AFC-131, TOPAS GmbH Company, Dresden, Germany). The samples were trimmed into a round shape with an area of 176.71 cm^2^. Diethylhexyl sebacate (DEHS) aerosol particles with a size of 0.218–4.595 µm and a flow velocity of 3.4 m^3^/h were used for the filtration test. The pressure drop resistance of the samples was measured with flow rates of 0, 0.7, 1.4, 2.0, 2.7, and 3.4 m^3^/h. The air permeability of PP and PP/PVA/ZIF-8 melt-blown electrospun composite membranes was measured at a pressure difference of 130 Pa by using a Full Automatic Air Permeability Meter (YG461H, Ningbo Textile Instrument Factory, Ningbo, China). The samples had an area of 176.71 cm^2^. Five samples for each specification were tested, and the results were averaged. The tensile properties of PP and PP/PVA/ZIF-8 melt-blown electrospun composite membranes were evaluated at a test rate of 200 ± 13 mm/min by using a Universal Strength Machine (5566, Instron Corporation, Norwood, MA, USA) as specified in American Society for Testing and Materials(ASTM) through tensile strength and elongation of fabric strip method ASTM D5035:1995(2003). The samples were trimmed into strips of 180 mm × 25.4 mm. The distance between clamps was 76 mm, and three samples for each specification were tested for the average. The filtration efficiency pressure drop and mean breaking strength were calculated using Equations (1)–(3), respectively [26,31,32].

(1) $$E=1-\left(\frac{C_{down}}{C_{up}}\right)$$

where *E* is the filtration efficiency, and *C_down_* and *C_up_* are the upstream and downstream aerosol concentrations, respectively.

(2) $$\Delta P=P_{1}-P_{2}$$

where Δ*P* is the pressure drop, *P*_1_ is the pressure before filtration, and *P*_2_ is the pressure after filtration.

(3) $$S_{A}=\frac{\Sigma_{i}^{n}S_{i}}{N_{n}}$$

where *S*_A_ is the mean breaking strength, *S*_i_ is the breaking strength of a specified sample each time, and *N*_n_ is the total number of samples for each specification. 

### 2.6. Characterization

A Fourier transform infrared (FT-IR) spectrometer (Nicolet iS10, Thermo Fisher Scientific, Waltham, MA, USA) was used to measure the permeation of ZIF-8 with wave numbers at 3750–250 cm^−1^. The functional groups of ZIF-8 were analyzed. ZIF-8 and kalii bromidum (KBr) were compressed at a ratio of 12:1. A FT-IR emissivity spectrum test was conducted. An X-ray diffraction (XRD) tester (D8 Advance, BRUKER, Karlsruhe, Baden-Württemberg, Germany) was used to scan ZIF-8 with parameters of 60 kV, 80 mA, and 5°–40°. A transmission electron microscope (TEM) (FEI Tecnai G2 Spirit TWINF, Hillsboro, OR, USA) was used to observe the surface morphology and interior structure of ZIF-8 at an accelerating voltage of 120 kV. The specific surface area and pore diameter of ZIF-8 were evaluated using a fully automatic specific surface and porosity analyzer (NOVA4200E, Anton Paar Kontha, Austria). Before the adsorption test, ZIF-8 weighing 0.3–0.4 g was subjected to deaeration with nitrogen and blown at 100 °C in an N_2_ environment for 5 h to remove vapor and purity. The specific surface area and pore diameter of ZIF-8 were analyzed according to the N_2_ gas adsorption–desorption isotherm. The micro-structures of PP melt-blown membranes and PP/PVA/ZIF-8 melt-blown electrospun composite membranes were observed at different magnifications by using a scanning electron microscope (SEM) (ZEISS Gemini SEM500, Oberkochen, Germany). The samples were pasted to a sample board with conductive glue and coated with a thin layer of gold. SEM was used to observe samples by using Image-Pro Plus6.0 image analysis software(Image-Pro Plus6.0, Media Cybernetics, Baltimore, MD, USA). Randomly selected 300 counts of fibers were measured to obtain the average diameter. The thermal stability of samples was assessed by a thermogravimetric analyzer (TGA) (G-209F3, NETZSCH Scientific Instruments Trading (Shanghai) Ltd., Shanghai, China). Samples weighing 10–15 mg were placed in a ceramic crucible and heated from 50 °C to 800 °C at increments of 25 °C/10 min in an N_2_ environment. Based on the TGA curves, the corresponding temperature for quality factor values of 95%, 90%, and 50% were studied to determine thermal stability.

## 3. Results and Discussion

### 3.1. Property and Characteristics of Synthesized ZIF-8

**Figure 2:**
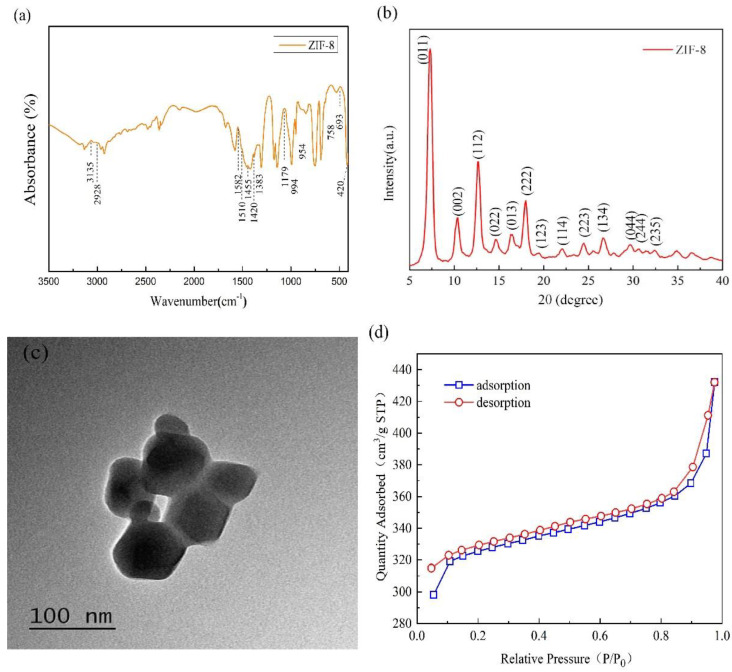
(**a**) Fourier transform infrared (FT-IR) spectrum, (**b**) X-ray diffraction (XRD) chart, (**c**) transmission electron microscopy (TEM) image, and (**d**) adsorption–desorption curves of ZIF-8.

Figure 2a shows the FT-IR spectrum of the synthesized ZIF-8 with characteristic bands at 3135, 2928, 1582,1510, 1455, 1420, 1383, 1179, 994, 954, 758, 693, and 420 cm^−1^ [33]. The stretching vibration of the Zn–N bond is presented at 420 cm^−1^; the stretching vibration or bending vibration of the imidazole ring is presented at 591–1550 cm^−1^; the stretching vibration of C=N in the imidazole ring is presented at 1580 cm^−1^; and the stretching vibration of C–H bonds for the imidazole ring in aromatic and aliphatic series is presented at 2930–3140 cm^−1^ [34,35]. The peak of the imidazole ring is presented at 1350–1550 cm^−1^; the peak of the hydroxyl group is presented at 2970 cm^−1^; the peak of free hydroxyl in the dissociative water molecule is presented at 3630 cm^−1^; and the peaks of N-H and O-H bonds are presented at 3070–3630 cm^−1^ [36]. The spectrum results are similar to the findings about ZIF-8 in previous studies [9].

Figure 2b shows the XRD chart of ZIF-8 and indicates two distinct spike-like peaks at 2θ = 7.3 and 2θ = 12.7, which suggest the high crystallinity of the crystal structure. In addition, the XRD pattern of ZIF-8 is consistent with that simulated by theoretic analogic computation. In Figure 2b, 2θ = 7.3, 10.4, 12.7, 14.7, 16.5, 18.0 and 19.5 have corresponding crystal faces (011), (002), (112), (022), (013), (222), (123), etc. Figure 2c shows the TEM chart of ZIF-8, where the composite exhibits regular and standard six-membered ring cell morphology but without large single crystals. ZIF-8 nanoparticles are prone to agglomeration, indicating the presence of some large particles formed by the agglomeration of trivial particles. Based on the analysis of ZIF-8 nanoparticles by using Image-Pro Plus6.0 software, the average particle diameter is 62 nm. The FT-IR spectrum, XRD, and TEM results indicate that the synthesized ZIF-8 has a correct structure and is substantiated to have sodalite (SOD) zeolite-type structure. The chemical formula of the synthesized composite ZIF-8 is shown in Figure 2b. The specific surface area of ZIF-8 is demonstrated in Figure 2d. ZIF-8 nanoparticles exhibit an N_2_ adsorption-desorption isotherm that is classified as type I curve. With low comparative pressure (P/Po < 0.02), the soaring adsorption is correlated with the micro-pore property of ZIF-8, indicating the presence of mesopores [27]. When P/Po is higher than 0.8, a hysteresis loop occurs and the second magnification shows medium/large porosity in the structure of ZIF-8. This result is attributed to the mesopores/macropores by the piled adjacent nanoparticles [27,35]. Hence, ZIF-8 nanoparticles are classified as mesoporous materials, and their structure implies excellent adsorption. Overall, ZIF-8 has a specific surface area of 1249.889 m^2^·g^−1^ and a pore diameter of 3.077 nm, which are consistent with previous findings [36]. As a result, ZIF-8 has a tremendous potential to adsorb PM_2.5_ particles due to its high specific surface area and mesoporous structure.

### 3.2. Surface Morphology of PP Melt-Blown Membrane and PP/PVA/ZIF-8 Melt-Blown Electrospun Composite Membranes

**Figure 3:**
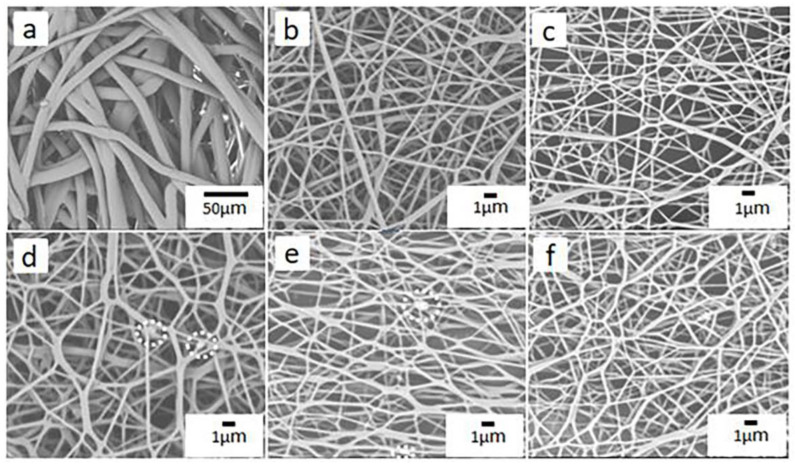
Scanning electron microscope (SEM) images of electrospinning: (**a**) PP melt-blown membranes, (**b**) PP/PVA, (**c**) PP/PVA/ZIF-8–1.25%, (**d**) PP/PVA/ZIF-8-2.5%, (**e**) PP/PVA/ZIF-8-3.75%, and (**f**) PP/PVA/ZIF-8-5%.

**Figure 4:**
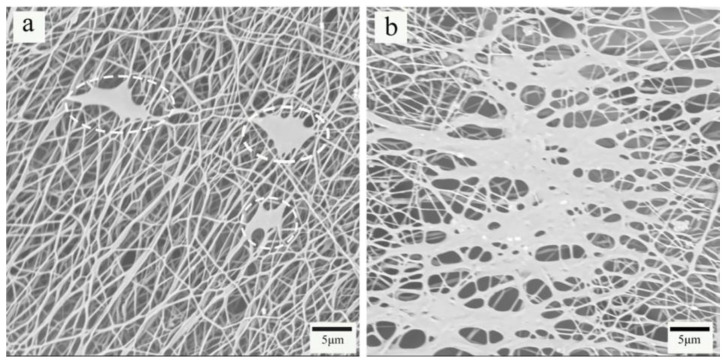
Agglomeration of (**a**) PP/PVA/ZIF-8-3.75% and (**b**) PP/PVA/ZIF-8-5%.

Figure 3 shows the SEM images of PP melt-blown membranes and PP/PVA/ZIF-8 melt-blown electrospun composite membranes. As shown in Figure 3a, the SEM image of PP melt-blown membranes at a magnification of 800 × shows that the fibers are thicker and smooth without splitting. The fibers demonstrate low entanglement, large pore size, low porosity, small web density, and uneven distribution. Figure 3b–e shows the electrospinning membrane of PP/PVA/ZIF-8 with different ZIF-8 content and the SEM images demonstrate that the fiber diameters of the electrospinning membrane are significantly reduced compared with the melt-blown membrane, and the entanglement among the fibers increase, which improves the porosity. With the increase of the ZIF-8 content, the aggregation of the ZIF-8 obvious, indicating ZIF-8 has been successfully loaded on the PVA/ZIF-8 electrospinning membranes. Figure 4a,b show the SEM images of electrospinning membrane that consists of 3.75% and 5% of ZIF-8, respectively. Large agglomeration areas are found among the fibers, and the area is proportional to the ZIF-8 content. 

**Figure 5:**
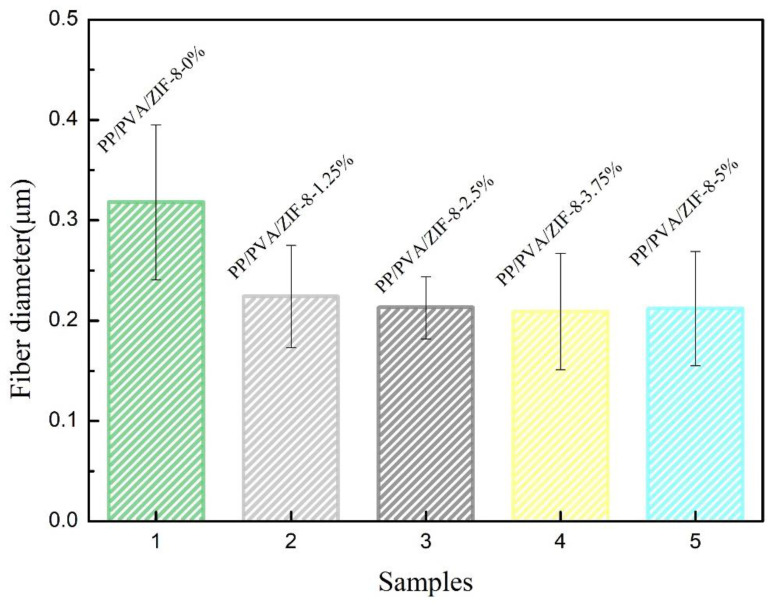
Average fiber diameter of PP melt-blown membranes and PP/PVA/ZIF-8 melt-blown electrospun composite membranes.

Figure 5 and Table S1 show the average diameter of electrospinning fibers. The use of electrospinning technology can directly reduce the average fiber fineness by about 64.7%, while the incorporation of ZIF-8 reduces the electrospinning fiber fineness. With ZIF-8 content of 3.75%, the average fiber diameter is 208.52 nm, which is the minimum value and is 34.4% lower than the average fiber diameter of PP/PVA melt-blown membranes. However, the average fiber diameter of PP/PVA/ZIF-8 melt-blown electrospun composite membranes is not proportional to the ZIF-8 content. An increase in the ZIF-8 content leads to an increasing trend in the fiber diameter because electrospinning fibrous membranes gradually become self-adhesive and form a continuous phase [37]. Severe particle aggregation still occurs in ZIFs-containing matrix membranes [38], and the specific surface area of PP/PVA/ZIF-8 melt-blown membranes increases with decreasing fiber diameter. A large specific surface area has a positive influence on the adsorption of nanoparticles over the fiber surface, thereby enhancing the filtration efficacy.

### 3.3. Filtration Performance of PP Melt-Blown Membranes and PP/PVA/ZIF-8 Melt-Blown Electrospun Composite Membranes

**Figure 6:**
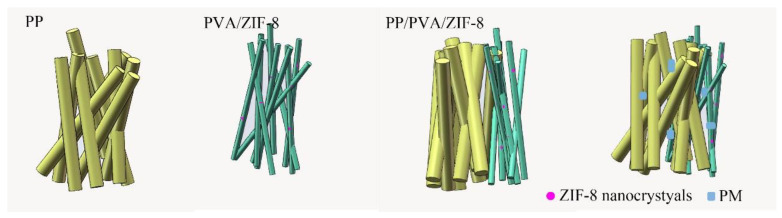
Filtration mechanism of PP/PVA/ZIF-8 melt-blown electrospun composite membranes.

The filtration mechanism of PP/PVA/ZIF-8 composite membranes is shown in Figure 6, where the upper layer is PP melt-blown membranes and the lower layer is ZIF-8-loaded PVA electrostatic spinning film. PM particles with large diameter are blocked by the first layer of filtration, and fine PM particles are blocked and adsorbed when passing through the second layer.

**Figure 7:**
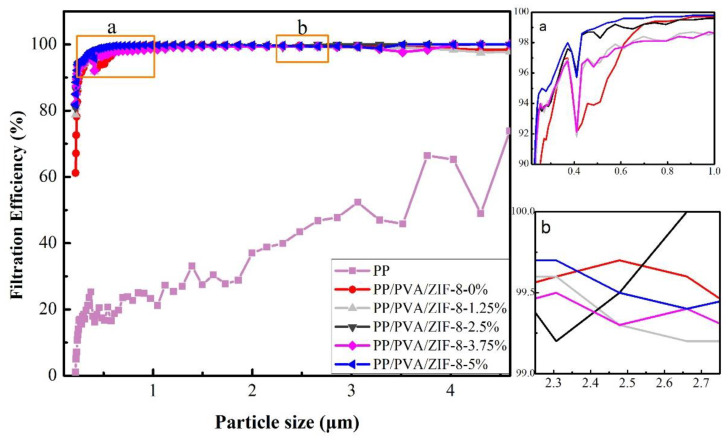
Filtration efficiency of PP melt-blown membranes and PP/PVA/ZIF-8 melt-blown electrospun composite membranes when diethylhexyl sebacate (DEHS) aerosol is 0.218-4.595 µm. (a) and (b) represent the enlarged part of the corresponding part in the figure.

Figure 7 shows the filtration efficiency of PP melt-blown membranes and PP/PVA/ZIF-8 melt-blown electrospun composite membranes with diethylhexyl sebacate (DEHS) aerosol (0.218–4.595 µm); the former exhibits distinctively lower filtration efficiency than the latter regardless of ZIF-8 content. PP melt-blown membranes cannot filter small particles because the constitutional fibers are thicker, resulting in large pore size, low porosity, and poor filtration efficiency. In the range of 2.661–4.595 mm, PP/PVA/ZIF-8-2.5% demonstrates 100% filtration efficiency and PP/PVA/ZIF-8-5% demonstrates 99.51% filtration efficiency. Electrospinning technology attenuates the fiber diameter of PVA/ZIF-8 membranes and aligns the fibers compactly, forming a ZIF-8-loaded electrospinning layer that features a delicate outlook and multiple micro-pores. With decreasing fiber diameter, the collection efficiency of the medium increases and the diameter associated with the most penetrating particles shifts toward lower values. Hence, the medium could fully intercept trivial particles that are not blocked by the melt-blown fabrics, thereby improving the filtration of trivial particles [39]. Figure S1 and Table S2 show the filtration efficiency of PP/PVA/ZIF-8 melt-blown electrospun composite membranes in relation to the DEHS aerosol (0.218–2.478 mm). The presence of ZIF-8 crystals enhances the average filtration efficiency. Furthermore, ZIF-8 has a considerable specific surface area, which facilitates the loading of ZIF-8 while increasing the pore volume and specific surface area. With 5% of ZIF-8, the membranes acquire the maximum filtration efficiency of 99.69 %, which is 5.79% greater than that of PP/PVA melt-blown electrospun composite membranes. Hence, PP/PVA/ZIF-8-5% melt-blown electrospun composite membranes demonstrate the highest filtration efficiency for PM_2.5_, followed by PP/PVA/ZIF-2.5% melt-blown electrospun composite membranes, because of the presence of ZIF-8 mesoporous materials. Mesoporous materials have a pore diameter that is between micropores and large pores and thus have a large specific surface area to attenuate fibers. With finer fibers, membranes can effectively intercept particles to acquire better adsorption.

**Figure 8:**
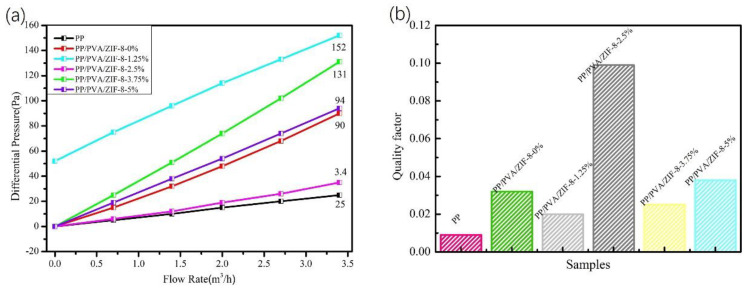
(**a**) Pressure drop resistance and (**b**) quality factor of membranes in relation to DEHS aerosol of 0.218–2.478 μm.

Figure 8a shows the pressure drop property of PP/PVA/ZIF-8 melt-blown electrospun composite membranes. According to Figure 8a, PP melt-blown membranes exhibit the lowest pressure drop resistance, which is lower than that of PP/PVA/ZIF-8 melt-blown electrospun composite membranes. Given that electrospinning fibers have smaller fiber diameter than the constituent fibers of melt-blown fabrics, the pores are densely packed. With respect to the loading amount of ZIF-8, PP/PVA/ZIF-8-2.5% melt-blown electrospun composite membranes exhibit the lowest pressure drop resistance. Nonetheless, the pressure drop resistance is not in proportion to the ZIF-8 content. Excessive ZIF-8 content in the electrospinning solution exacerbates the agglomeration of fibers, leading to few pores formed. To sum up, ZIF-8 content of 2.5% effectively reduces the pressure drop resistance of PP/PVA/ZIF-8 melt-blown electrospun composite membranes.

The average filtration efficiency and pressure drop resistance of PP/PVA/ZIF-8 melt-blown electrospun composite membranes in relation to DEHS aerosol are compared and summarized. The addition of ZIF-8 crystals significantly improves the filtration efficiency but adversely affects the pressure drop resistance. Filtration efficiency and pressure drop resistance are two essential factors for air filters. Therefore, quality factor (*Q_f_*) is incorporated to examine the filtration performance of filters and is computed using the following Equation [40].

(4) $$Q_{f}=-\frac{\ln\left(1-\eta \right)}{\Delta P}$$

where *η* is the filtration efficiency (%), and ∆*P* is the pressure drop resistance (Pa).

Hence, the quality factor of PP/PVA/ZIF-8 melt-blown electrospun composite membranes is dependent on filtration efficiency and pressure drop resistance. The quality factor is high when the filtration efficiency is high and the pressure drop resistance is low, suggesting that PP/PVA/ZIF-8 melt-blown electrospun composite membranes have filtration performance that is proportional to the quality factor. The greater the quality factor is, the better the filtration performance will be. Therefore, quality factor can indicate the filtration performance of PP/PVA/ZIF-8 melt-blown electrospun composite membranes. The quality factors of PP melt-blown membranes and PP/PVA/ZIF-8 melt-blown electrospun composite membranes containing different ZIF-8 contents with DEHS aerosol (0.218–2.47 μm) are shown in Table 5, Figure 8b, and Table S3. Figure 8b shows that PP/PVA/ZIF-8 melt-blown electrospun composite membranes have greater quality factor than PP melt-blown membranes. Hence, the use of electrospinning technology enhances the PM_2.5_ filtration performance of the materials. By contrast, the quality factors of traditional melt-blown non-woven fabrics are 0.036 and 0.038 when the filtration efficiencies are 94.29% and 96.63%, respectively. In the present study, PP/PVA/ZIF-8-2.5% acquires an average of 96.5% filtration efficiency for PM_2.5_ and has a quality factor of 0.099, which is 2.61 times that of traditional melt-blown membranes and 3 times that of PP/PVA membranes. The quality factor is not proportional to ZIF-8 content, so PP/PVA/ZIF-8-2.5% membranes outperform the rest of the membranes in terms of filtration performance for PM_2.5_ and PM_5_ particles. 

**Figure 9:**
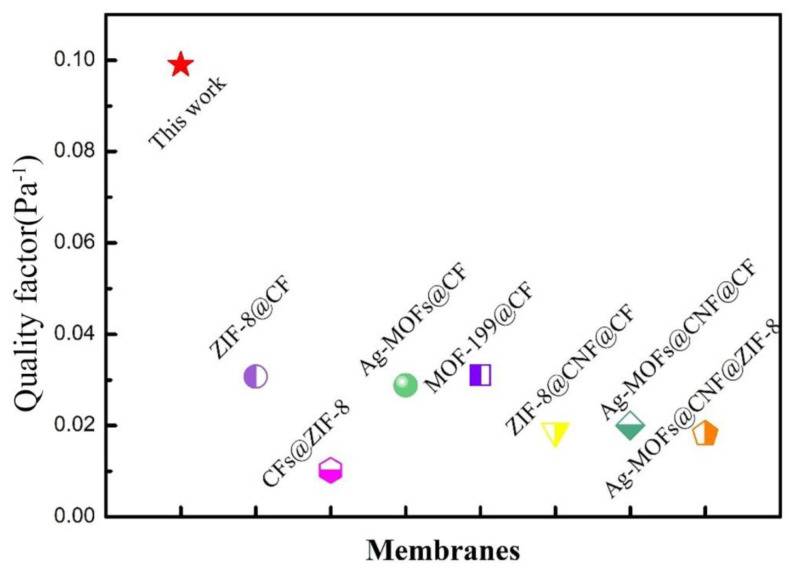
Quality factor of PP/PVA/ZIF-8-2.5 % melt-blown electrospun composite membranes and other air filters.

Figure 9 and Table S4 show the comparison of the quality factor of PP/PVA/ZIF-8-2.5% membrane with other filters at the same airflow rate. The PP/PVA/ZIF-8-2.5% membrane has an excellent PM_2.5_ filtration performance, which is better than that of ZIF-8 and MOF-loaded material reported so far. PP/PVA/ZIF-8-2.5% membrane has excellent double membranes structure, different layers can filter particles of different diameter of particles. ZIF-8 not only has a large specific surface area, it can also refine the electrospun fiber and improve the overall filtration efficiency and adsorption capacity. It is speculated that the pores between the two membranes may help retain particles, and these effects synergistically lead to a higher quality factor.

### 3.4. Thermogravimetric Analyzer (TGA) and Mechanical Properties of PP Melt-Blown Membranes and PP/PVA/ZIF-8 Melt-Blown Electrospun Composite Membranes

**Figure 10:**
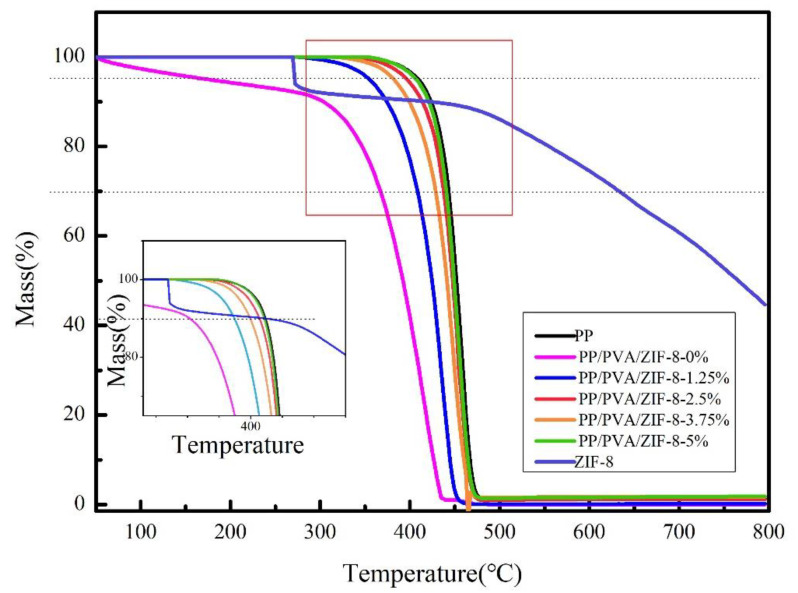
Thermogravimetric (TG) curves of PP melt-blown membranes and PP/PVA/ZIF-8 melt-blown electrospun composite membranes.

PP is a thermoplastic material with excellent heat resistance. The melting point of PP melt-blown membranes reaches 176 °C. PVA possesses good biocompatibility and can be decomposed in nature, but it demonstrates poor thermal stability. ZIF-8 possesses excellent thermal and chemical stability. Figure 10 shows that PP melt-blown membranes have the maximum thermal stability, but the addition of PVA significantly decreases the thermal stability of PP/PVA membranes. PP/PVA membranes also display weight loss after the temperature of 50 °C, and the weight loss exacerbates at 300 °C–425 °C. 

The TG curve confirms that ZIF-8 possesses excellent thermal and chemical stability. An increasing proportion of ZIF-8 crystals have a positive influence on the thermal stability of PP/PVA/ZIF-8 melt-blown electrospun composite membranes, and both have a proportional relationship that compensates for the interference of PVA. In addition, high ZIF-8 content delays the initial decomposition temperature of the filter membranes, which substantiates the positive influence of ZIF-8 on thermal stability. A descending gradient is presented, indicating that ZIF-8 is compatible with other components. As such, ZIF-8 crystals are loaded over PVA/ZIF-8 membranes successfully. In particular, PP/PVA/ZIF-8-5% membranes exhibit the maximum thermal stability; at 407.6 °C and 423.5 °C, the quality factor values are 95% and 90%, respectively; these temperatures are 57.2% and 39.45% higher than those of PP/PVA. The presence of ZIF-8 prevents hazards exerted by high temperatures (Table S5). 

**Figure 11:**
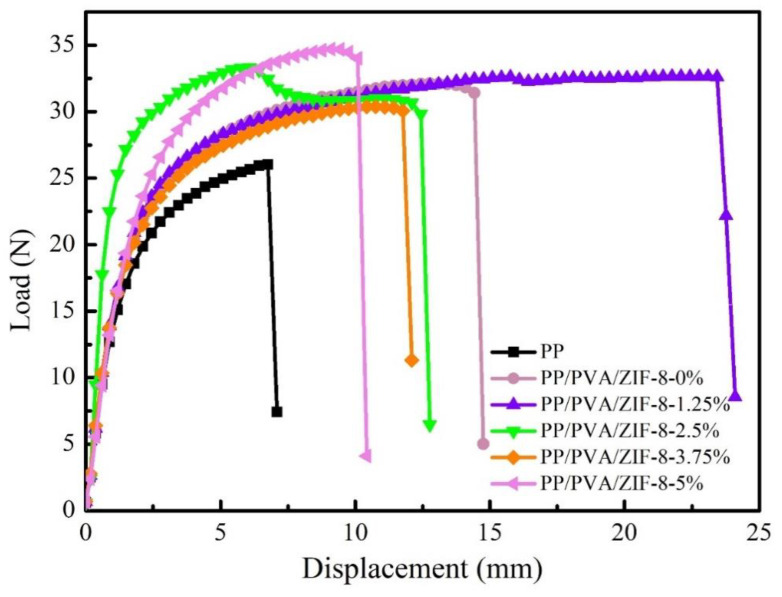
Displacement-Load curves of PP melt-blown membranes and PP/PVA/ZIF-8 melt-blown electrospun composite membranes.

**Figure 12:**
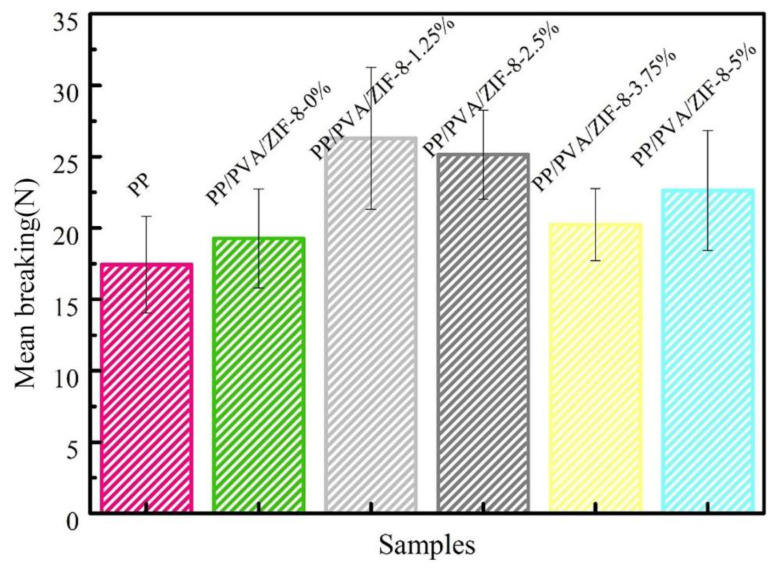
Mean breaking strength of PP melt-blown membranes and PP/PVA/ZIF-8 melt-blown electrospun composite membranes.

The displacement-load and the mean breaking strength of PP melt-blown membranes and PP/PVA/ZIF-8 melt-blown electrospun composite membranes are presented in Figure 11 and Figure 12. As shown in Figure 12, PP melt blown membranes have the lowest mean breaking strength given that all PP/PVA/ZIF-8 melt-blown electrospun composite membranes have high average breaking strength regardless of ZIF-8 content. This result is due to the fact that electrospinning technology coats a nanofiber membrane over PP melt-blown membranes. Electrospinning generates ultra-fine fibers that exhibit high friction and good fiber cohesion. As a result, the components of the filter membrane have a synergized interaction; the membranes exhibit greater tensile strength at break. Meanwhile, PP/PVA/ZIF-8-1.25% exhibits the highest mean breaking strength, followed by PP/PVA/ZIF-8-2.5% among PP/PVA/ZIF-8 melt-blown electrospun composite membranes containing different ZIF-8 contents. The mean breaking strength of PP/PVA/ZIF-8 melt-blown electrospun composite membranes has no proportional relationship to ZIF-8 content. An increase in the breaking strength suggests efficient load transfer after pure PP melt-blown membranes are loaded with ZIF-8 particles. Sufficient load transfer is attributed to even distribution of ZIF-8 in the matrices and the good interfacial adhesion between PVA and ZIF-8 nanoparticles [41]. 

### 3.5. Air Permeability of PP Melt-Blown Membranes and PP/PVA/ZIF-8 Melt-Blown Electrospun Composite Membranes

**Figure 13:**
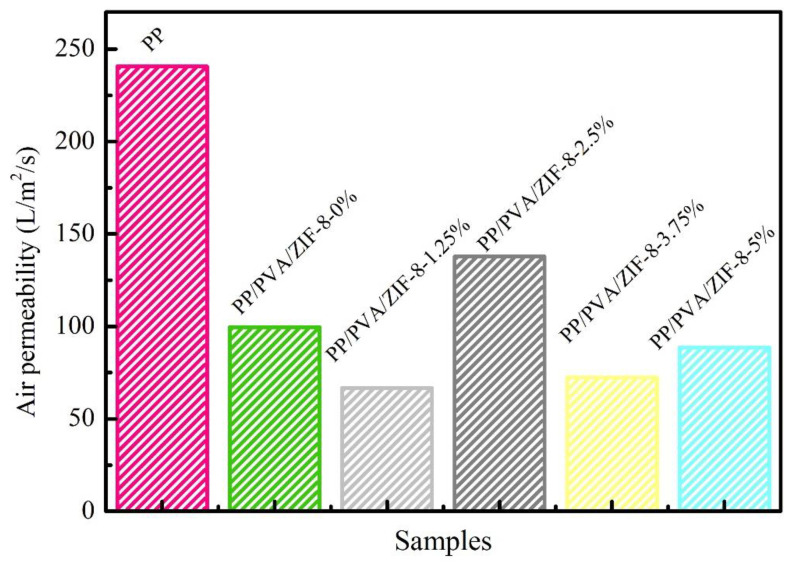
Air permeability of PP melt-blown membranes and PP/PVA/ZIF-8 melt-blown electrospun composite membranes.

Figure 13 and Table S6 show the air permeability of PP melt-blown membranes and PP/PVA/ZIF-8 melt-blown electrospun composite membranes. PP melt-blown membranes exhibit the highest air permeability, whereas PP/PVA/ZIF-8 melt-blown electrospun composite membranes have lower air permeability. The nanofiber membrane coated over PP melt-blown membranes by electrospinning adversely affects the air permeability. In comparison with the air permeability of different PP/PVA/ZIF-8 melt-blown electrospun composite membranes, PP/PVA/ZIF-8-2.5% exhibits the maximum air permeability, which is 38% higher than that of PP/PVA/ZIF-0%. This result is directly associated with the fact that PP/PVA/ZIF-2.5% has the lowest pressure drop resistance, and air permeability is not proportional to ZIF-8 content. Based on the analysis of pressure drop resistance and air permeability of PP melt-blown membranes and PP/PVA/ZIF-8 melt-blown electrospun composite membranes, the pressure drop resistance is inversely proportional to air permeability. The higher the air permeability is, the greater the comfortable texture will be. Hence, PP/PVA/ZIF-8-2.5% membranes have favorable filtration efficiency and comfortable texture. 

## 4. Conclusions

In this study, melt-blown and electrospun techniques are used to produce metallic frame-based PP/PVA/ZIF-8 melt-blown/electrospun membranes. The filtration performance of the membranes is studied and compared with other melt-blown filters and electrospun membranes. PP/PVA/ZIF-8 melt-blown/electrospun membranes are proven to yield the optimal mechanical properties of melt-blown materials as well as good filtration performance of electrospun membranes. Furthermore, ZIF-8 is successfully loaded over PP/PVA melt-blown electrospun composite membranes to form PP/PVA/ZIF-8 melt-blown electrospun membranes, which are qualified as an air filter. The test results indicate that the proposed composite membranes have significantly high breaking strength, with values of 26.3 and 25.1 N when the ZIF-8 contents are 1.25% and 2.5%, respectively. Moreover, the breaking strength is higher than that of pure PP membranes. In addition, the presence of ZIF-8 distinctively improves the filtration efficiency of filter membranes. With 2.5% of ZIF-8, the composite membranes show good air permeability, high mechanical properties, and optimal PM_2.5_ filtration performance, which is 96.5% with a quality factor that is 11 times that of pure PP melt-blown membranes and 3 times that of PP/PVA membranes. With the benefit of an economical production cost, PP/PVA/ZIF-8-2.5% melt-blown/electrospun membranes have great application prospects in the PM_2.5_ air filter field.

## Figures and Tables

**Table 1:** Temperatures required for melt blowing.

Screw 1 (°C)	Screw 2 (°C)	Screw 3 (°C)	Pipeline (°C)	Metering Pump (°C)	Die (°C)	Hot Air Duct Temperature (°C)
180	280	310	310	200	193	210

**Table 2:** Parameters of melt blowing.

Screw Pressure (Mpa)	Metering Pump Flow (r·min^−1^)	Air Pressure (MPa)	Collector Speed (cm·min^−1^)	Distance/cm
0.1–1	7.6	0.03	44	20

**Table 3:** Parameters of electrospinning process.

Pump-Flow (mL/h)	Pump-Area (mm^2^)	Drum Diameter (mm)	Winding Speed (mm/s)	Round Trip Distance (mm)
0.8	187.62	19.11	150.00	120.00
Moving Speed (mm/s)	Reduction Ratio	Voltage (kV)	Current (mA)	
30.00	1.00	24.00	0.01	

**Table 4:** Denotations and compositions of PP/PVA/ZIF-8 melt-blown electrospun composite membranes.

Sample	Ingredient
PP/PVA	PVA(10%)ZIF-8(0%)
PP/PVA/ZIF-8-1.25%	PVA(10%)ZIF-8(1.25%)
PP/PVA/ZIF-8-2.5%	PVA(10%)ZIF-8(2.5%)
PP/PVA/ZIF-83.75%	PVA(10%)ZIF-8(3.75%)
PP/PVA/ZIF-8-5%	PVA(10%)ZIF-8(5%)

**Table 5:** Average filtration efficiency, pressure drop resistance, and quality factor of PP melt-blown membranes and PP/PVA/ZIF-8 melt-blown electrospun composite membranes.

Sample	PM (μm)	Average Filtration Efficiency (%)	Pressure Drop Resistance (Pa)	Quality Factor
PP	0.218–2.478	21.0	24	0.009
	0.218–4.595	26.5		0.013
PP/PVA	0.218–2.478	93.8	88	0.032
	0.218–4.595	94.6		0.033
PP/PVA/ZIF-8-1.25%	0.218–2.478	95.6	152	0.020
	0.218–4.595	96.1		0.021
PP/PVA/ZIF-8-2.5%	0.218–2.478	96.5	34	0.099
	0.218–4.595	97.1		0.103
PP/PVA/ZIF-8-3.75%	0.218–2.478	95.9	130	0.025
	0.218–4.595	96.4		0.026
PP/PVA/ZIF-8-5%	0.218–2.478	97.1	94	0.038
	0.218–4.595	97.4		0.039

## Supplementary Materials

The following are available online at https://www.mdpi.com/2079-4991/10/10/2025/s1, Figure S1: Average filtration efficiency of PP melt-blown membranes and PP/PVA/ZIF-8 melt-blown electrospun composite membranes when DEHS aerosol is 0.218-2.478 µm, Table S1: Average fiber diameter of PP melt-blown membranes and PP/PVA/ZIF-8 membranes; Table S2: Average filtration efficiency of PP melt-blown membranes and PP/PVA/ZIF-8 membranes when DEHS aerosol is 0.218-2.478 µm; Table S3: Quality factor of membranes as related to the DEHS aerosol being 0.218-2.478 μm; Table S4: Comparison of comprehensive PM_2.5_ filtration performance of PP/PVA/ZIF-8-2.5% melt-blown electrospun composite membranes and other air filters; Table S5: Temperatures required by PP melt-blown membranes and PP/PVA/ZIF-8 melt-blown electrospun composite membranes as related to 90% and 50%; Table S6: Air permeability of PP melt-blown membranes and PP/PVA/ZIF-8 melt-blown electrospun composite membranesm.
