# Supplementary material for: Polypropylene/Polyvinyl Alcohol/Metal-Organic Framework-Based Melt-Blown Electrospun Composite Membranes for Highly Efficient Filtration of PM2.5
Source: Nanomaterials (Basel). 2020 Oct 14;10(10):2025. doi: 10.3390/nano10102025 (PMC7602219; doi:10.3390/nano10102025)
Supplement: Supplementary file 1 [file nanomaterials-10-02025-s001.pdf]

Supporting Information

Polypropylene/Polyvinyl Alcohol/ Metal–Organic Framework Based Melt-Blown Electrospun Composite Membranes for Highly Efficient Filtration of $PM_{2.5}$

Table S1. Average fiber diameter of PP melt-blown membranes and PP/PVA/ZIF-8 membranes.

Membranes	PP/PVA/ZIF-8-0%	PP/PVA/ZIF-8-1.25%	PP/PVA/ZIF-8-2.5%	PP/PVA/ZIF-8-3.75%	PP/PVA/ZIF-8-5%
average fiber diameter(μm)	0.318±0.077	0.224±0.051	0.213±0.031	0.209±0.058	0.212±0.057

Table S2. Average filtration efficiency of PP melt-blown membranes and PP/PVA/ZIF-8 membranes when DEHS aerosol is 0.218-2.478 μm.

Membranes	PP	PP/PVA/ZIF-8-0%	PP/PVA/ZIF-8-1.25%	PP/PVA/ZIF-8-2.5%	PP/PVA/ZIF-8-3.75%	PP/PVA/ZIF-8-5%
average filtration efficiency (%)	21	93.8	95.6	96.5	95.9	97.1

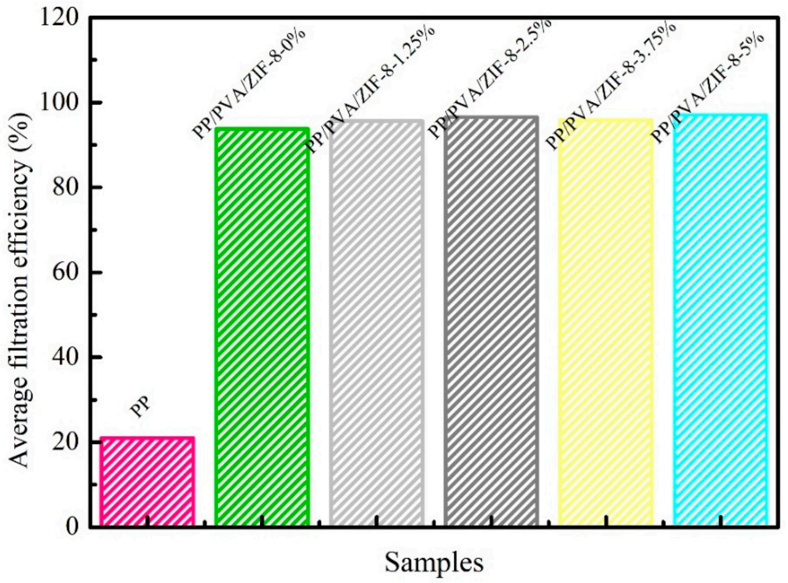

Figure S1. Average filtration efficiency of PP melt-blown membranes and PP/PVA/ZIF-8 melt-blown electrospun composite membranes when DEHS aerosol is 0.218-2.478 μm.

Table S3. Quality factor of membranes as related to the DEHS aerosol being 0.218-2.478 μm.

Membranes	PP	PP/PVA/ZIF-8-0%	PP/PVA/ZIF-8-1.25%	PP/PVA/ZIF-8-2.5%	PP/PVA/ZIF-8-3.75%	PP/PVA/ZIF-8-5%
average filtration efficiency (Pa^{-1})	0.009	0.032	0.02	0.099	0.025	0.038

Table S4. Comparison of comprehensive $PM_{2.5}$ filtration performance of PP/PVA/ZIF-8-2.5% melt-blown electrospun composite membranes and other air filters.

Membranes	PM(μm)	E_a (%)	ΔP_b(Pa)	Qf_c (Pa-1)	Ref
PP/PVA/ZIF-8-2.5%	DEHS	96.5	34.00	0.099	This work
ZIF-8@CF	DEHS	98.36	134.00	0.0307	2018[7]
CFs@ZIF-8	DEHS	99.90	680.50	0.0102	2018[6]
Ag-MOFs@CF	DEHS	97.34	126.00	0.0288	2018[7]
MOF-199@CF	DEHS	98.28	131.00	0.0310	2018[7]
ZIF-8@CNF@CF	DEHS	93.75	148.00	0.0187	2019[5]
Ag-MOFs@CNF@CF	DEHS	91.25	122.00	0.0200	2019[5]
Ag-MOFs@CNF@ZIF-8	DEHS	94.30	157.81	0.0182	2019[5]

[a] $PM_{2.5}$filtration efficiency. [b] Pressure drop. [c] Quality factor.

Table S5. Temperatures required by PP melt-blown membranes and PP/PVA/ZIF-8 melt-blown electrospun composite membranes as related to 90% and 50%.

Sample	Temperature at a mass fraction of 95% (℃)	Temperature at a mass fraction of 90% (℃)	Temperature at a mass fraction of 50%(℃)
PP/PVA	174.4	303.7	392.2
PP/PVA/ZIF-8-1.25 %	355.2	381.6	431.9
PP/PVA/ZIF-8-2.5 %	397.7	404.8	445.0
PP/PVA/ZIF-8-3.75 %	382.8	419.2	454.0
PP/PVA/ZIF-8- 5%	407.6	423.5	451.7

Table S6. Air permeability of PP melt-blown membranes and PP/PVA/ZIF-8 melt-blown electrospun composite membranesm .

Membranes	PP	PP/PVA/ZIF-8-0%	PP/PVA/ZIF-8-1.25%	PP/PVA/ZIF-8-2.5%	PP/PVA/ZIF-8-3.75%	PP/PVA/ZIF-8-5%
Air permeability ($L/m^2/s$)	240.62	99.60	66.81	137.92	72.44	88.71
